# Children’s insights into nutritional culture and habits in Abu Dhabi

**DOI:** 10.3389/fpubh.2026.1746039

**Published:** 2026-03-26

**Authors:** Preetha Menon, Muhammad Uba Abdulazeez, Mouza Salem Alnuaimi, Maryam Humaid Alnaaimi, Omar Mohamed Alzaabi, Shama Humaid Almeqbaali, Dana Mubarak Aljneibi, Saoud Faraj Altamimi, Fatima Sultan Alsaedi, Hamad Ali Alshehhi, Messaouda Belfakir, Salam Omar, Mohamed El-Sadig, Aminu Abdullahi, Syed Shah, Kassim Abdullah

**Affiliations:** 1 Institute of Public Health, College of Medicine and Health Sciences, United Arab Emirates University, Al Ain, United Arab Emirates; 2 Zayed Centre for Health Sciences, United Arab Emirates University, Al Ain, United Arab Emirates; 3 Department of Education, Collage of Arts, Education and Social Sciences, Abu Dhabi University, Abu Dhabi, United Arab Emirates

**Keywords:** food, nutrition, children, Abu Dhabi, focus group discussion

## Abstract

**Introduction:**

The United Arab Emirates (UAE) has identified childhood obesity as a significant public health concern. Designing targeted interventions to promote healthy eating habits in early childhood requires an understanding of the food preferences and norms within this age group. The present study aimed to describe the unique food culture and influences among schoolchildren in Abu Dhabi Emirate (AD), UAE.

**Methods:**

We conducted a qualitative study based on the grounded theory approach. Eleven bilingual focus group discussions (FGDs) were conducted involving 64 children from nursery to grade school (aged 4–8 years) based on theoretical sampling. The transcripts were axial coded iteratively by multiple researchers.

**Results:**

Four themes emerged from the FGDs with the children. First, children’s awareness of healthy food begins early in nursery school and is reinforced by their teachers and peers. Second, school lunchbox norms and food policies strongly influenced children’s food choices. Third, school food choice is heavily influenced by preparation time. Fourth, cultural norms around food aesthetics limit many healthy food options.

**Discussion:**

School food culture and practices in early childhood are constantly influenced by various systems, including family, school, peers, and the community. These systems significantly impact children’s food preferences, awareness of healthy eating, and ultimately their school food habits. Understanding these interconnected systems are crucial for creating supportive environments that foster healthy food habits during early childhood in AD and the UAE as a whole.

## 1 Introduction

Childhood overweight and obesity represents a major public health issue due to its association with the development of chronic diseases. Excess weight in children is a contributing factor to the onset of health related conditions such as type two diabetes, cardiovascular diseases, and other long-term health problems that can continue into adulthood (1). Given the increasing prevalence of childhood overweight and obesity worldwide, it is imperative to explore the factors that drive unhealthy eating habits and sedentary behaviors in children (2).

The global trend of rising childhood overweight and obesity is prevalent in the Middle Eastern region as well (3–5). Research has indicated a growing prevalence of overweight and obesity in preschool and elementary schoolchildren (3). The United Arab Emirates (UAE) is no exception, with several studies reporting the very high prevalence of childhood overweight and obesity, ranging from 20 to 38% (6–16). These figures significantly exceeds the global average of 8% (17). This calls for an early intervention to reduce overweight and obesity in this age group in the country.

This public health challenge in the Gulf Cooperation Council countries, including the UAE, is characterized by rapid economic development and the adoption of Western dietary patterns (18). Understanding the food culture and dietary habits of children in a specific region is crucial for addressing childhood overweight and obesity (19). Early childhood food culture is especially important because dietary habits formed during this period can persist throughout life and contribute to the risk of obesity and other diseases.

Several studies have investigated obesogenic dietary practices among children, particularly adolescents, using quantitative methods (20–22). These studies have shown that among pre-adolescents, schools and parents have a greater influence on food choices compared to peers or social media (21, 23–28). This makes schools a powerful environment for implementing long-lasting behavioral change interventions. The school environment can influence early childhood eating habits through factors such as food availability, advertising, peer influences, and nutritional education (25, 27, 29–31). These factors can shape children’s food preferences and consumption patterns (29, 32). Additionally, it has been observed that the influence of schools on early childhood eating habits varies depending on factors such as school’s resources, community’s socioeconomic status, and cultural norms (25, 33).

Numerous studies conducted in the Middle East have also explored dietary habits in early childhood (21, 22, 34, 35). However, there is a lack of studies investigating cultural influences, particularly concerning school food culture. Additionally, while these studies have described the eating culture, they did not provide insights into the socio-cultural factors that sustain these habits. This qualitative study involving children’s perspectives tried to fill this gap by describing the unique food culture among schoolchildren in Abu Dhabi Emirate (AD). We delve further to understand the unique social norms and influences on food preferences among children in the emirate. This is imperative for designing targeted interventions to promote healthy eating habits among children.

## 2 Materials and methods

### 2.1 Research objective and study design

Food habits developed in early childhood have a lasting health impact throughout life. Therefore, there is a need for deeper insights into how children perceive and engage with food, especially their school meals. The present study was based on classic grounded theory outlined by Glaser and Strauss (36) while the analysis followed the procedures of open coding, axial coding, and coding framework proposed by Strauss and Corbin (37). This approach is ideal for revealing deep-rooted insights from the children’s perceptions. This method brings to light multiple factors that are beyond the cognitive ability of young children.

### 2.2 Research tool development

A comprehensive literature review as performed to develop the initial focus group discussion (FGD) guide. This informed the choice of the initial questions and topics. A flexible approach was employed whereby the responses from the children guide the direction of the subsequent questions. The resulting semi-structured FGD guide was validated by qualitative research experts, cultural specialists, and child development professionals to ensure its relevance and effectiveness. This helped to modify the FGD protocol by introducing picture-based activities at the beginning of each session to stimulate the initial discussions and serve as a basis for deeper conversations regarding the study’s topics. Keeping the target children’s short attention span in mind, the FGD was designed around group activities based on the research questions. The discussions and activity-based questions were shortened to minimize fatigue identified during a pilot test with six-year-old children.

### 2.3 Participants

We conducted eleven FGDs involving 64 children from nursery school (aged four years), kindergarten (aged five to six years), and grade school (aged seven to eight years). All the selected children participated in the discussions. These children typically spend between five to seven hours in school, five days a week. While nursery and kindergarten schools encourage food prepared from home, grade schools have limited options in school canteens. A diverse group of children were selected from private and public schools across the three regions of AD (Al Dhafra, Abu Dhabi, Al Ain). Table 1 presents the demographic characteristics of the children involved in the study.

**Table 1:** Demographic characteristics of the children involved in the study.

FGD	Date	Region	School	Ethnicity	Age^*^	Gender
1	11/10/2023	Al Ain	Public	Emirati	7–8	3 boys, 3 girls
2	11/10/2023	Al Ain	Public	Emirati	7–8	3 boys, 3 girls
3	18/10/2023	Al Ain	Public	Emirati	7–8	3 boys, 3 girls
4	20/02/2024	Al Ain	Private	Arab, African, South Asian	4–5	1 boy, 4 girls
5	21/02/2024	Al Ain	Private	South Asian	7–8	3 boys, 4 girls
Data analysis: first round of coding						
6	13/05/2024	Al Dhafra	Private	Arab	7–8	2 boys, 4 girls
7	20/05/2024	Abu Dhabi	Private	Arab	7–8	4 boys, 2 girls
8	22/05/2024	Abu Dhabi	Private	Arab	7–8	1 boy, 5 girls
9	27/05/2024	Abu Dhabi	Public	Emirati	4–5	4 boys, 2 girls
10	27/05/2024	Abu Dhabi	Public	Emirati	5–6	1 boy, 4 girls
11	29/05/2024	Abu Dhabi	Public	Emirati	5–6	3 boys, 2 girls
Data analysis: second round of coding						

^*^Years.

### 2.4 Theoretical sampling

We included a representative sample of children from public and private schools in Al Ain, Abu Dhabi, and Al Dhafra regions of AD. Children from various nationalities representing the ethnic configuration of the emirate participated in this study. Some FGDs were delayed and conducted after the initial round of coding. While the coders were not involved in the data collection, the moderator was. This unintentional theoretical sampling allowed for further exploration and clarification of concepts that were vague in the initial analysis. The emergent codes guided the moderators’ questions thereby leading to more detailed discussions regarding the cultural influences and barriers to healthy nutritional habits among the children.

### 2.5 Data collection

The FGDs were conducted with a mixed group of boys and girls of the same age. These discussions took place in quiet environments, such as the school nurse’s office or designated meeting rooms. The moderators were introduced to the children by the study facilitator (class teacher or school nurse) and the study was explained to the children by the moderators before commencing each FGD. Two trained moderators fluent in both English and Arabic engaged the children with picture activity-based questions and prompts. To ensure balanced participation among the children, prior to commencing every FGD, the moderators established the rule of not interrupting any speaker and waiting until the person speaking has completed talking before the next participant can speak. The children’s responses influenced the moderator’s prompts, leading to additional detailed discussions and insights into cultural influences and barriers to healthy nutritional habits among the children. The discussions were audio recorded and each session lasted for twenty to thirty minutes. The moderators were selected from a group of trained researchers and had no prior contact or relationship with the children. The FGDs were conducted during the period of October 2023 to May 2024.

### 2.6 Research team

The research team was headed by a researcher with over fifteen years of experience in qualitative research. To ensure quality and consistency in data collection, twelve researchers were trained comprehensively in qualitative research. Their training sessions included qualitative study approach, FGD process, ethical considerations when conducting research involving children and the study’s topics. Four researchers helped develop the study tool, performed its cultural validation and moderated the FGDs while the other eight researchers performed the qualitative data analysis using the “NVivo” software (Lumivero, Denver, United States). This includes the transcription, familiarization, blinded coding, and coding tree development.

### 2.7 Ethical consideration

This study was approved by the Social Sciences Research Ethics Committee of UAE University (ERSC_2023_2507). Additionally, approval for the study was obtained from the Abu Dhabi Education Department (for private schools) and Emirates Schools Establishment (for public schools). Parental written informed consent was obtained before data collection and only the children that assented to participate as well as having parental approval were involved. The children were free to leave and rejoin the discussion as they wish.

To protect the children’s identities, fictional names or pseudonyms based on their favorite fruit or animation character were used in the audio recordings. Any inadvertently recorded identifiers were removed from the transcripts. The researchers and moderators avoided taking videos and photographs. The FGDs were conducted in quiet rooms without the presence of schoolteachers. No incentives were given to the children, their parents, or the school staff/authorities. The discussion transcripts were not shared with the children, their parents or school authorities.

### 2.8 Reflexivity and bias

The researchers who analyzed the transcripts were not involved in the data collection. A reflexivity exercise was conducted among the eight researchers trained for analysis. Their individual responses to the FGD questions were discussed to identify the dominant cultural beliefs and biases. These beliefs were challenged within the group, resulting in debates that used personal examples. The coders’ self-reflections were reassessed between the first and the second rounds of coding. If the emergent codes resembled the researcher’s perceptions, the team examined the contributing participant quotes for bias.

### 2.9 Data analysis

The audio recordings were transcribed using AI tool (Turboscribe) but required corrections and translations when necessary. Multiple blinded coding was performed by the researchers who first familiarized themselves with the transcript and then generated codes based on their interpretation. Multiple coders analyzed each transcript to ensure consistent interpretation. We did not use *a priori* codes and opted for open coding. We held three meetings to discuss the meaning and rationale behind the diverse emergent codes. A few researchers grouped these codes into related themes following the protocol for axial coding. These groupings were challenged by the rest of the group members through multiple sessions to arrive at a concise and coherent framework. We reached a consensus on the code definitions and categories after an iterative process to form a coding framework. This codebook was then applied to the same transcripts for focused coding.

Line-by-line coding was done using NVivo software version 14 (Lumivero, Denver, United States). The resulting codes from each researcher were exported to Excel sheets before the synthesis. The synthesis and the summarization of selected core categories central to the conceptual framework took place twice, following two phases of data collection (Table 1). These categories were subsequently reorganized to align with the emerging theoretical framework. The emerging themes were summarized and visualized using the word clouds graphical representation. Ultimately, the grounded theory analysis approach culminated in organizing these findings into a coherent narrative.

## 3 Results

### 3.1 Description of school lunchbox contents

#### 3.1.1 Typical food items in school lunchboxes

**Figure 1:**
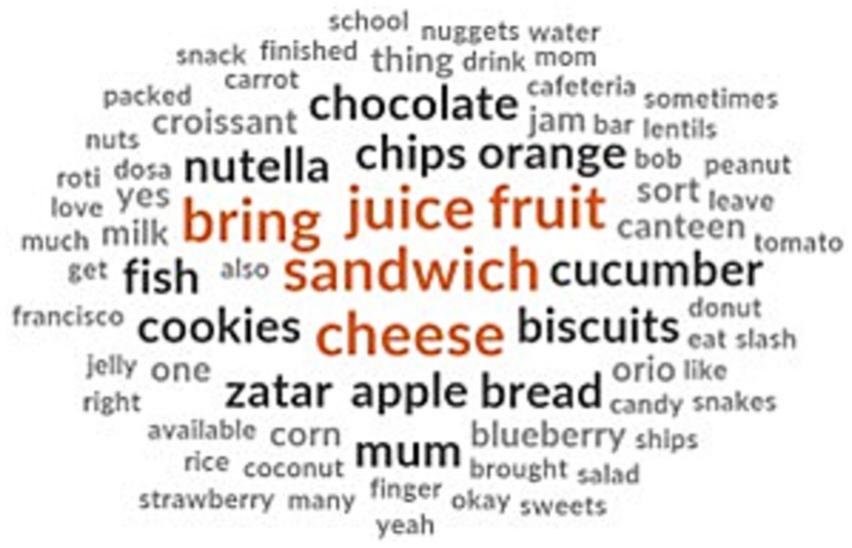
School lunchbox menu identified by the children.

The children listed the food items they usually bring to school which consisted of homemade snacks like sandwiches with cheese, Nutella and croissants (Figure 1). They also mentioned fruits and fruit juices as one of the frequent items in their lunchbox. Among fruits, the children brought apples, oranges, blueberries and strawberries to school. Baked and fried snacks like chips, cookies, Oreos, croissants and biscuits were also in the lunchbox menu in addition to raw salads like cucumber, carrot and tomato. The children also mentioned bringing nuts, peanuts, candies, chocolate, Nutella spread and jam as sweet supplements to their school meal. Cooked meals included unleavened bread (roti), lentils and fish.

#### 3.1.2 Excluded food items

**Figure 2:**
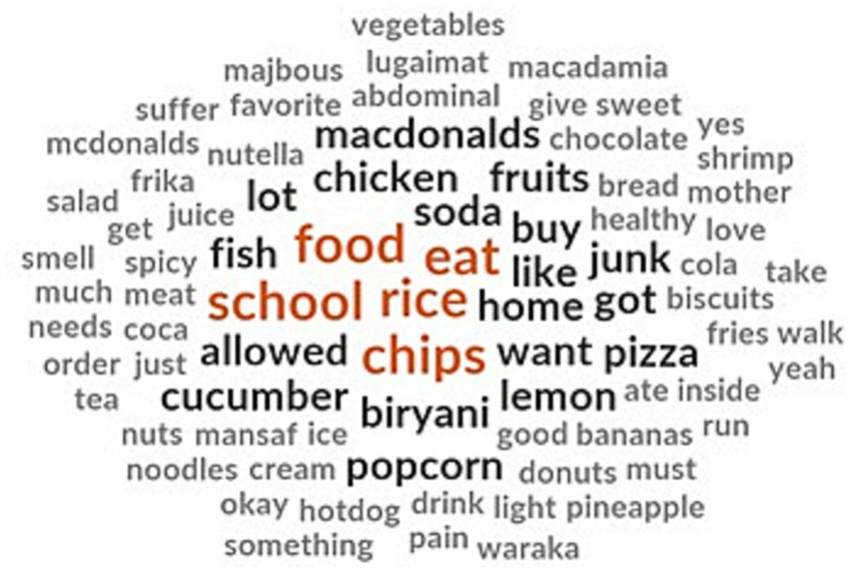
Children’s responses on their favorite but excluded food items from school meal.

The children mentioned a few of their favorite food items that they do not bring to school. They reported not bringing traditional foods like rice, biriyani, Frika, Waraka, Machbous and Mansaf to school. Other favorite foods like noodles, fish, shrimp and chicken were not included in the school lunchbox (Figure 2). This is despite the children liking to eat these foods at home. They mentioned avoiding chips, French fries, cookies, pizza, McDonald’s meals, donuts, Coca-Cola and soda in their lunchboxes in compliance with the school policy discouraging these foods and drinks. At the same time, some school cafeteria provides chips, croissants, sandwiches and juices as alternatives to homemade food.

### 3.2 Factors influencing school food culture and habits

The best-suited thematic framework model for these themes is Bronfenbrenner’s Ecological Systems Theory which examines how different environmental factors influence individual behaviors and development (38). This model is ideal for understanding the multifaceted factors affecting the children’s school food culture and habits. It incorporates the interplay between individual, familial, cultural, institutional, and societal influences.

#### 3.2.1 School food culture and habit microsystem influences

To understand the school food culture, it was important to elucidate the impact of the immediate environment surrounding children, including their family, school, peers, and neighborhood. These are the most direct influences that affect children on a daily basis, on what they eat and how they eat which may directly impact their daily experiences with food.

##### 3.2.1.1 Personal preferences and eating aid

The children mentioned food preferences as a reason for not bringing healthy meals to school. They prefer sweet or salty snacks that they can finish at school. Their personal preferences for visually appealing and easy-to-eat snacks were expressed for both the school and home meals. Some children reason that they need to watch TV to eat certain food at home and therefore, they will not eat it at school.

*“I like chips because Crunch crunch crunch. It tastes good …Because it’s salty It’s salty” (Nursery, Expatriate, Al Ain)*

##### 3.2.1.2 Children’s healthy food awareness

**Figure 3:**
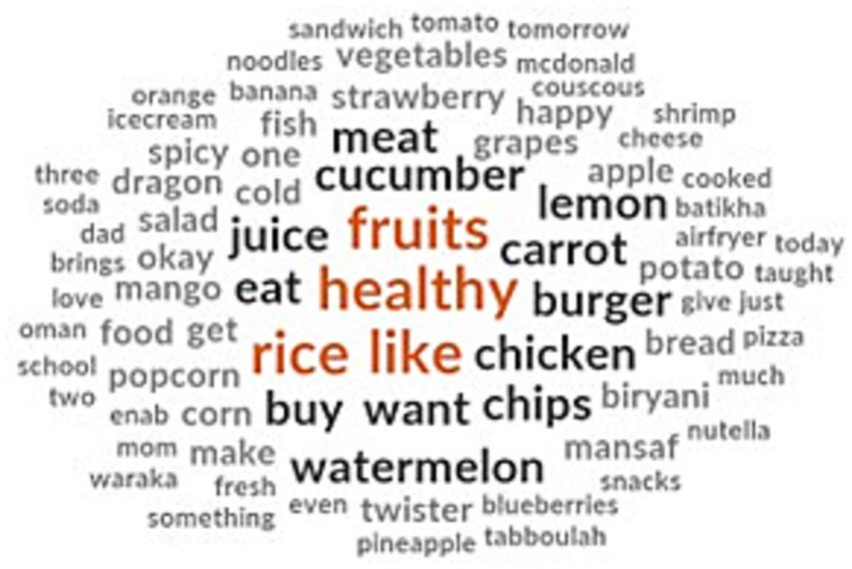
Children’s healthy food perceptions.

The FGDs gave an insight into what the children considered healthy food and junk food. They categorized fruits like strawberries, blueberries, oranges, watermelon, grapes and mangoes as healthy food. They also considered cucumber, carrot, raw salad, tabbouleh and traditional food such as rice, couscous and Waraka as healthy (Figure 3). Additionally, sandwich, cheese and home-cooked foods were identified as healthy. In contrast, some nursery children identified chips, Mc Donald’s meals and burgers as healthy food. The children identified Pepsi and soda as unhealthy food. Some children even labelled fish, cucumber and apples as unhealthy food.

##### 3.2.1.3 School curriculum

Schools include awareness of a balanced and healthy diet in the first term of every academic year in nurseries and kindergarten. The FGDs revealed the awareness of the children that certain foods like fish and fruits are beneficial to the body and need to be consumed frequently. In addition, they identified food items like carrots and grapevine as being responsible for good vision. These messages are drilled in classes and reinforced through activities like no-fire cooking festivals and food charts. The children differentiated between the food that is allowed, the food that is discouraged, and the food that is good for their health, irrespective of their preferences. Their understanding of what constitutes healthy and unhealthy food is influenced by school discussions and policies.

##### 3.2.1.4 Policy implementation through inspection and reminders

The teachers’ involvement in monitoring school lunchbox contents reinforces the school food policies and communicates it to parents. Awareness of these food-related policies was spread through class teachers, school sessions on healthy food, reminder notices on the school gates, school food festivals with parents, and parent communications.

*“Because it is written on the door—not allowed.” (Kindergarten, Emirati, Abu Dhabi)*

*“We have a wall in the class, and we get minus points on the wall when we bring junk.” (Grade school, Expatriate, Al Dhafra)*

##### 3.2.1.5 Peer influence on healthy eating

The influence of other children in the school can encourage or discourage certain food choices. In some schools, peer influence discouraged children from bringing chips or soda to school. The children reported eating chips and drinking soda at home but refraining from doing so in schools due to peer influence.

##### 3.2.1.6 Food aesthetics and peer norms

Eating healthy meals as school lunch has many barriers. One of the most cited reasons by the children for not eating healthy home-cooked meals has been food aesthetics and storage issues. Healthy food options that the children like to eat at home such as fish, Waraka and rice dishes were not brought to school due to their unpleasant or strong smell. The children reported avoiding strong smelling food in the classroom; a norm followed in most classrooms. Some children equate strong smelling food to eating spoilt food and falling sick. In addition, the children prefer not bringing vegetables or other home meals as they are not visually attractive. While they like to eat these foods at home, they cite reasons like lingering smell in their hands and not having enough soap to wash their hands after the meal as rationales for not including such foods in their school lunchbox.

*“Smell will spread. it is not smells good. Waraka is smell. Fish is smell.” (Kindergarten, Emirati, Abu Dhabi)*

##### 3.2.1.7 Family roles in school food preparation

**Figure 4:**
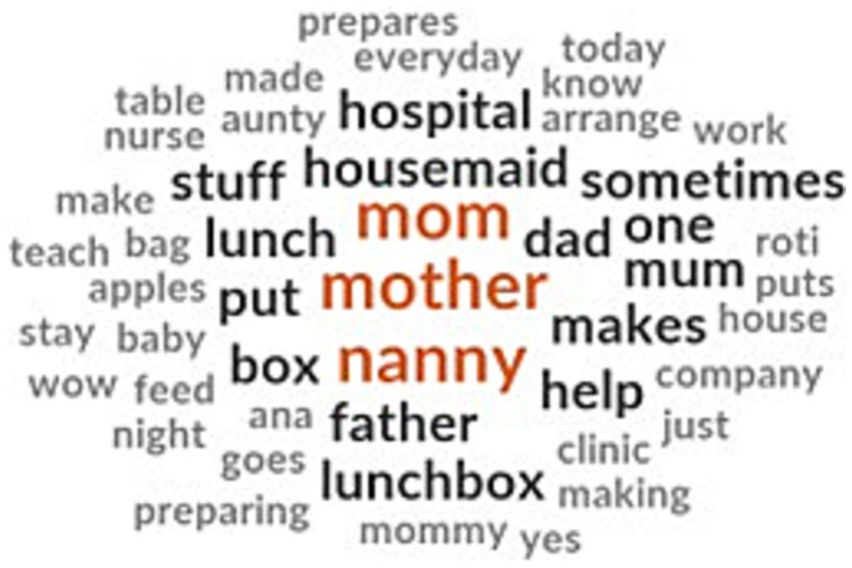
Family members involved in preparing school food.

Since almost all the children brought food from home, we asked them regarding who prepared their school lunchboxes and who had a say in deciding the lunchbox menu. Preparing snacks and fruits in the lunchboxes was predominantly done by the child’s mother or nanny (Figure 4). In some households, fathers help in preparing the lunchboxes while some children prepare their lunchboxes themselves by either putting together processed food or helping their mothers in the process. In some families, other members like aunts pitched in when their mothers were busy or sick.

#### 3.2.2 School lunch culture and habit mesosystem influence

Unlike the previous direct influences that children experience themselves, the following influences hint at the interaction between the different stakeholders like the influence of the school on parents’ choices.

##### 3.2.2.1 Impact of school food policy

This connects the school’s environment (microsystem) with the home environment (another microsystem). Unhealthy or junk food is a concept taught to children in school and reinforced by school food policies. The children acknowledged the school policy of encouraging home-prepared food. While a few schools allowed processed and semi-processed food like biscuits and croissants, some schools barred them. The schools were strict in enforcing the food policy including homemade food and fresh fruits exclusively. Even candies and cakes were allowed only once a year during the children’s birthdays.

##### 3.2.2.2 Early education and community

Categorizing food as “allowed,” “discouraged,” or “good for health,” based on school teachings and peer influence starts right from the first term in nursery school. This concept of permissible and disallowed food is drilled early at school which is passed on to family members during school activities like food week and food festivals. ‘Healthy’ food competitions among parents or children in these events help in disseminating these concepts to the larger community.

##### 3.2.2.3 Restriction on sharing school lunch

In all schools, sharing school lunch among children was discouraged to avoid ingesting food allergens. These policies are strictly enforced and the children mentioned that their teachers stopped them from sharing their food with other classmates.

##### 3.2.2.4 Communication between schools and parents

The school’s policies directly influences what the parents prepare for their children and the schools have reinforced these policies through direct communications with parents. The school policy of encouraging healthy food is enforced through regular food inspections by the class teachers during breaktime. Children bringing high energy and processed foods like chips are identified and their parents were notified and reminded of the school food policy.

##### 3.2.2.5 Family decision-making dynamics

While mothers and nannies are involved in preparing the school lunchbox, deciding the contents of the lunchboxes lies with the mothers and fathers. In some cases, older siblings decide the menu. The children sometimes requested their parents for certain favorite foods. It could be understood from the children’s responses that some parents permitted processed and calory rich food like chocolates, chips and cookies while others did not. While some parents complied with the school food policy and did not allow junk food in the school lunchbox, some parents allowed their children to eat calorie rich snacks in the house whereas others allowed them in limited quantities.

##### 3.2.2.6 Time constraints in school meal preparation

Time constraints to healthy meal preparation have been reported by the children. They cite the inability of housemaids or mothers to cook healthy meals early in the morning, as it is a time-consuming process. Some children observed that home-cooked meals take an hour or more to prepare with the resulting extra work on housemaids or nannies. Some children cite younger siblings and babies in the family as the reason why their mothers are unable to cook healthy foods for their school meals early in the morning.

*“All the healthy food takes time to make, and it's difficult to make it in the morning, right?” (Kindergarten, Emirati, Abu Dhabi)*

##### 3.2.2.7 Innovations to improve the tastiness of healthy foods

The children gave suggestions like food modifications that make healthy food attractive enough for school lunch menu. They suggested increasing the visual attractiveness of school lunch by including vegetables and fruits of different colors. They called it ‘rainbow salad’ and included fruits of different colors. Some children that are very picky with food color mentioned eating ‘pink’ fruits for lunch. Some children from a school that discouraged processed food came up with cooking strategies that allowed tasty and healthy school lunch meals. Certain foods like soups and Pulav were declared tasty even when it turned cold by lunchtime. Some children suggested cooking food early in the morning or having the provision of heating their school lunch to encourage warm meals. Adding vegetables to rice in the form of Pulav and biriyani was a favored healthy home-cooked school lunch menu highlighted by many South Asian children. Some children suggested adding fruits to ice cream to make it healthy. In addition, they suggested cooked meals in the school cafeteria instead of the usual options like processed food and juice. Cold sandwiches are the norm in school cafeterias and some children suggested adding vegetables to cheese to make it healthier.

#### 3.2.3 School food culture and habit ecosystem influences

In addition to exploring the direct influences on the children’s school lunch experience, the FGDs revealed insights into other external influences at the school and education policy level. These influences were observed throughout the discussions at different schools. These are local government policies that reflect certain food choices and habits.

##### 3.2.3.1 School cafeteria options

This reflects the broader availability and accessibility of healthy food options in the school. While the cafeteria was absent in some schools, other schools had limited snack options in the school cafeteria. The children reported access to the school cafeteria for snacks like sandwiches, shawarma, burger, croissants, juices, cookies and chips. In schools without a cafeteria, the children were limited to eat food brought from home.

##### 3.2.3.2 Food storage and reheating infrastructure

Food storage was one of the most frequently cited reason by the children for not bringing home-cooked meals or other healthy food options to school. The fear of spilling food in the bag or food leakage was a commonly cited reason for not bringing healthy meals and preferring dry snacks in the school lunchbox. The children cited the lack of storage and food reheating facilities (microwaves) as a reason why they do not bring food that could spoil quickly. Cold food being unappetizing was one of the reasons why the children avoided healthy food cooked in their homes. The children commented that they would eat healthy meals if they were freshly cooked and hot. However, these options are not available in the school cafeteria as mentioned by the children.

##### 3.2.3.3 Impact of short lunchbreaks

Limited lunch breaks of fifteen to forty minutes have influenced the children’s food choices. The children complained that healthy meals need more time to eat. They shared their challenge in trying to wash their hands and eat meals within the limited lunch break. They also prefer eating fast foods as it gives them time to play or interact with their friends during lunch breaks. Some children cite the fear of abdominal pain if they eat heavy meals in school.

*“Ah, it takes a long time to make rice, it takes too long to eat rice, right?” (Kindergarten, Emirati, Abu Dhabi)*

*“No, we have only ten minutes, ten minutes for lunch break, we get five minutes maximum for eating, five minutes maximum, to go, wash your hands, come back, from to the washroom”(Grade school, Expatriate, Al Ain)*

##### 3.2.3.4 Impact of the national school health program

The school nurses include healthy diet communications to the parents of overweight and obese children. These children were identified following the school screening programs conducted at the schools.

#### 3.2.4 School lunch culture and habit macrosystem influences

This section delves into the societal norms, cultural values and belief systems identified in the FGDs which have the potential to influence school food habits. These norms influenced the type of food the children were exposed to at home and their overall perception of eating. These cultural norms became evident when comparing the responses of the children from different cultural backgrounds, namely Emirati, Arab, and South Asian.

##### 3.2.4.1 Cultural differences in school lunch preferences

**Figure 5:**
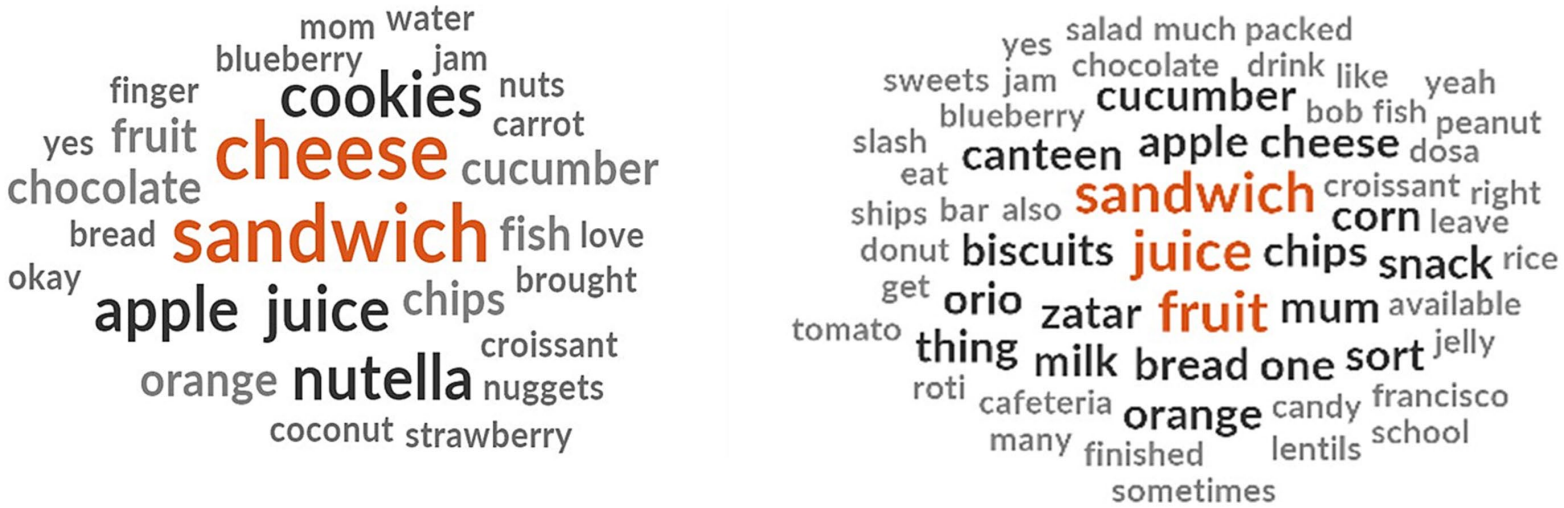
Preferred school lunch menu of Emirati children (left) compared to South Asian children (right).

Responses regarding the school lunch menu were different between Emirati and expatriate children. Emirati children brought sandwiches, cheese, cookies, fruits and fruit juices as the predominant food items in their school lunchbox. In addition, to these options, South Asian children included homecooked meals like Biriyani, Pulav, lentils and roti (Figure 5).

##### 3.2.4.2 Cultural norms shaping family involvement in school lunch preparation

**Figure 6:**
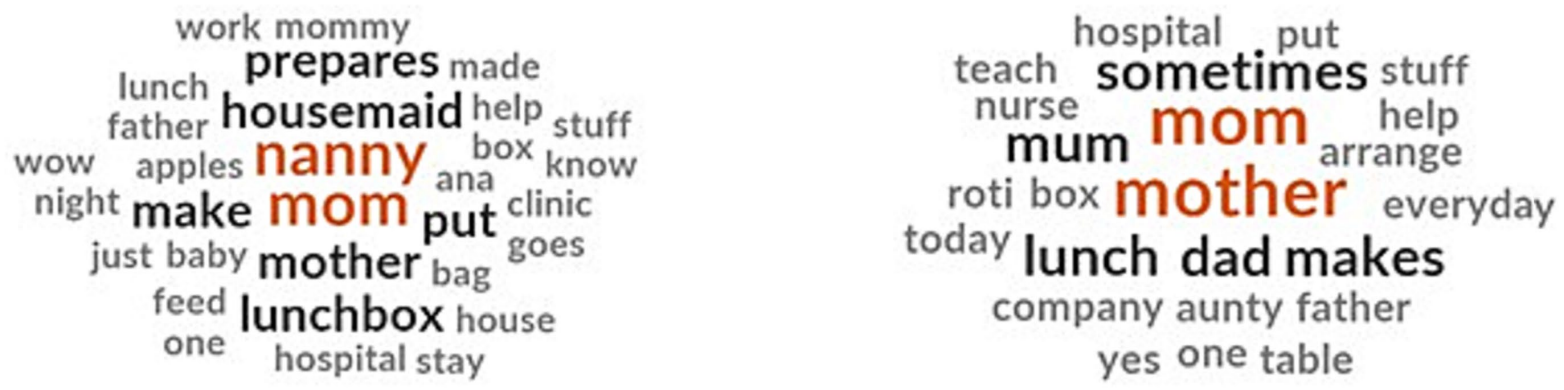
Family members involved in preparing school lunch for Emirati children (left) and expatriate children (right).

There were differences between Emirati and expatriate children when it comes to the person who prepared their school lunchboxes. For Emirati children, housemaids and nannies had a dominant role in preparing the lunchbox, in addition to mothers as well as the children themselves (Figure 6). Fathers were never mentioned in the household task of preparing or deciding the school lunch menu. In expatriate households, the role of mothers in preparing school lunchboxes dominated the discussions. Fathers also contributed to preparing the lunchbox. The children themselves preparing their school lunchbox was not mentioned in the FGDs among expatriate children.

##### 3.2.4.3 Perception of school as a study environment

Cultural beliefs that a heavy lunch is not suitable for school emerged from some of the FGDs. Some children observed that their parents avoided heavy meals and packed snacks in their school lunchbox in the belief that heavy meals could give rise to abdominal pain. This was more so when the children played during or after lunch breaks. Some children mentioned their elder siblings’ comments that the school is a place of study, with a focus on academic activities. They dichotomized play and food from academics, with heavy meals being delegated to the home environment.

*“We must take light food to school so when we walk or run, we won’t suffer of abdominal pain”. (Grade school, Expatriate, Al Dhafra)*

**Figure 7:**
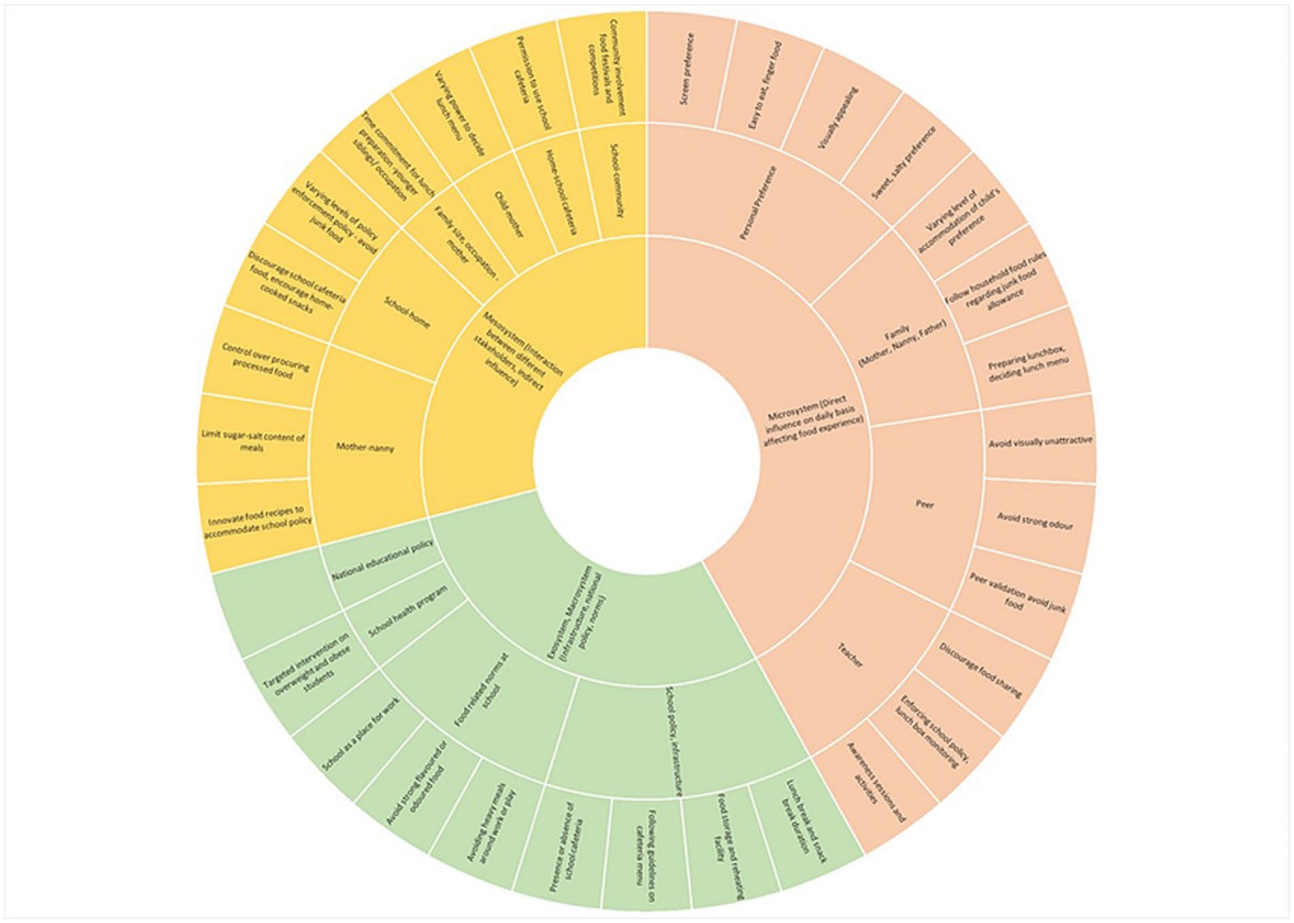
Healthy eating influences for children in Abu Dhabi emirate.

The general overview of the several influences of healthy nutrition for school children in AD is presented in Figure 7. The figure illustrates the theoretical framework employed in the current study by showing the multifaceted influences shaping eating habits among young children in AD. The model depicts a complex interplay of factors, categorized for clarity, that contribute to children’s food choices and overall eating behaviors.

## 4 Discussion

The current study observed that the awareness of healthy food among school children in AD begins early in nursery school, with teachers and peers reinforcing it through lunch box norms and school policies. There is a significant variation in school lunch policies and their influence on parents’ food choices. Family involvement in lunchbox preparation, primarily by mothers and nannies, is heavily influenced by school policies and limited preparation time. Cultural norms around food aesthetics limit many healthy food options, further compounded by a lack of infrastructure to store and reheat food at schools. Additionally, limited time for eating meals is a major barrier to a healthy lunch experience for schoolchildren.

### 4.1 Children’s awareness of healthy food

The findings of the present study show that incorporating food literacy into early childhood education (nurseries and kindergartens) improves children’s awareness of healthy diets. This is consistent with a randomized controlled trial which demonstrated that a targeted nutrition education curriculum positively impacts young children’s dietary habits, nutrition knowledge, and overall health. Results from this short, six week intervention reported significantly higher scores on nutrition and health and showed greater preferences for fruits and vegetables (30). Moreover, a concept mapping study involving Dutch primary schoolchildren, concluded that children have a wide variety of perceptions of what is important for their health and wellbeing (39). The children in the current study showed awareness similar to Portuguese preschoolers with a five-week educational intervention (40). We hypothesize that this awareness will promote vegetable consumption as reported in the aforementioned study on Portuguese preschoolers.

### 4.2 Cultural influences and social diffusion

School meals are important not only physically and nutritionally, but they contain deep cultural and social aspects as well, often involving religious practices and proscriptions (41). In the UAE, similar to other countries in the Middle East, due to globalization and the diversified population, the nutritional culture has been influenced by international cuisines, and this has had an impact on every aspect of local food culture (18, 20, 22, 35). However, we noticed a difference between the food culture and cuisine at home and at school similar to observations from previous studies in the UAE and Saudi Arabia (21, 42–44). This hints at a distinct non-traditional, aesthetic food culture at school, influenced by school food guidelines. Studies and interventions have shown how school meal plans can be challenging in a multi-cultural environment like the UAE (45, 46).

Despite these differences, school environments still play a significant role in shaping children’s food culture and eating habits (47). Research among preschool children showed that both the physical and social environment influence eating patterns (35). Pre-adolescents growing up in environments consuming less junk-food ended up with healthier eating habits (23, 32, 33, 48). This influence stems from both official school policies, peers, teachers, and parents (23, 24, 26).

The school environment has an impact on the food culture of children and the community, and this impact may last for many generations. This influence goes beyond academic development, as schools also act as social hubs, fostering the development of social skills, sense of belonging and recognizing acceptable behavior (32, 47, 49, 50). In schools, the food culture develops through observational learning, and children adhere to food-related norms, which are often reinforced by peer influence (40, 42, 51–53). In addition, food socialization and peer conformation promote food acceptance and diet diversity even among picky eaters (22, 47, 54). The present study observed children opting for healthier lunch choices, avoiding energy-dense drinks and processed foods in school, as a desire for social belonging. This supports the idea that peer influence plays a strong role in shaping food choices within the school setting (55).

The healthy eating habits fostered at school are not just confined to the school walls; they spread outward into the wider community. The present study found that schoolchildren are actively involving their families and communities in healthy eating initiatives. The children reported organizing “Healthy Food Week” events and “Healthy Chef Competitions” with their families and neighbors, supporting the evidence regarding how school-based healthy eating practices are diffusing beyond the school setting (56). Transforming food culture at schools involves multiple stakeholders including schoolchildren, teachers, school administrators, cafeteria managers, local food suppliers, families and other actors in the community where the schools are located (52). Such school coordinated efforts engaging the community may spill over its benefits beyond formal food education.

The food customs and practices learned at school can become part of a family’s traditions, passed down to the next generations. The current study observed how the simple cheese sandwich, a staple school lunch, has been passed down from parents to their children. This transgenerational adoption of a non-traditional food option (for many cultures) highlights the long-term impact of food norms influenced by schools. Parents influence their children’s food habits by controlling their food availability, food menu, mealtime routine introducing food diversity, reinforcing food rules, and acting as role models (57). Food choices of parents both for home meals and school lunch influences the food choices the children make when they start a family (54). It can be concluded that the positive impact of school-based nutrition interventions can be passed down to future generations (21, 28). In other words, the healthy habits learned at schools can ripple out to impact the entire community over time.

### 4.3 Increasing the impact of school policies towards healthier diet

The present study revealed that national policies in the UAE promote healthy eating habits in schools. These policies include guidelines for school lunch, incorporating food literacy into the curriculum, and a school health program focused on promoting healthy habits among overweight and obese schoolchildren. The school environment plays a critical and direct role in influencing eating behaviors, especially in the impressionable early childhood age (27, 58, 59). Policies that restrict energy dense low nutritional food in school meals and school cafeterias have been proven to influence children’s nutritional intake (25, 32, 52, 55). This influence could be direct by familiarizing schoolchildren with healthier food options in reimbursable school meals as seen in schools around poor neighbourhoods in New Jersey (49). Interventions like the Smart Lunch Boxes in the United Kingdom has successfully influenced parents in adopting healthier school lunch options by increasing the portion size of fruits, vegetables, and dairy leading to a direct increase in Vitamin A and folate levels among the schoolchildren (60).

The UAE launched a national taskforce in 2022 to develop a roadmap for tackling the childhood obesity crisis in the country (61). The taskforce consists of different stakeholders from government agencies (Dubai Health Authority, Ministry of Health and Prevention, Abu Dhabi Public Health Centre, and Department of Health), private institutions, policymakers, food manufacturers and suppliers, individuals, schools, and the media. The findings of the current study supports the utility of such a taskforce based on the different levels of the Ecological Model in tackling the menace of childhood obesity in the UAE. Additionally, the findings of the present study demonstrates the need for the involvement of additional stakeholders in the taskforce in the form of social actors, civil society, community members, and academic groups for the successful implementation of nutritional policies to address the prevalence of childhood obesity in the country.

A study on food-based guidelines in schools showed that countries like the UAE have guidelines for schools, but not for nurseries (4). The school guidelines have clear recommendations on the dietary requirement for different age groups and permitted foods in the school cafeteria (4, 46). However, there is no evidence regarding whether these guidelines and food literacy are diffused to the households that prepare the school lunch for the children. Thus, the older school children who eat school cafeteria meals might be benefiting from these guidelines (30, 34, 46). However, younger elementary school and nursery children seem to be beyond the influence of these guidelines, which are shared with school authorities and not parents. This is a policy influenced situation as nurseries do not provide school lunch meals and food is expected to be brought from home (62).

The current study shows that preparing snacks and fruits in the school lunchboxes was predominantly done by the children’s mothers or nannies. In some households, fathers helped in preparing the lunchboxes with the help of the children themselves. This highlights the influence of stakeholders like parents in improving school lunch. Studies have proven that early childhood nutrition is greatly supported by the nutritional literacy of the parents (63). This inter-generational influence, albeit weaker, is also observed among adolescents (64). These findings were employed in the SMART lunch box intervention towards improving food literacy with clear guidelines to parents. This intervention successfully led to an improvement in the food and nutritional content of children’s school lunch (60).

The present study observed differences between Emirati and expatriate children with regards to their school lunch menu. This finding aligns with previous studies that explored the influence of the diverse home food environment and its correlates on eating behaviors and school lunch in the UAE (20, 21, 44). These findings highlight the need to consider cultural diversity when designing child healthy nutritional interventions targeted towards parents in the UAE. School meal guidelines are targeted towards school authorities while the nutritional literacy curriculum is targeted towards the schoolchildren. These interventions have created school food norms, which encourage healthy food options and discourage unhealthy food consumption. Curriculum-based instructions on food literacy in terms of recognizing healthy food and junk food is known to have a strong influence on young children, especially in kindergarten (29, 30, 64, 65). The current study observed an increased awareness level and self-efficacy being reinforced by social norms of avoiding junk food in schools. Sadly, the desire to fit in and be accepted by their peers sometimes leads children to avoid healthy foods that have strong smells or do not look appealing. The present study has shown that schools have the power to influence food norms among young children and hence needs to extend its influence on healthier but less attractive food.

Healthier food options must be supported by a conducive environment. The children in the current study reported that they do not have enough time to eat their school lunch with greater priority to socialising and active play. School lunch was considered as a social event involving chatting and playing, leaving very little time for eating (45, 47, 50, 53). A longer school lunch period has been suggested by previous studies that observed an increase in school lunch time resulted in increased consumption of fruits and vegetables by schoolchildren (33, 50). Thus, the nutritional advantage of healthy food at school is compounded by a healthier eating habit which would be further reinforced as a norm among school children.

### 4.4 Future research

Future studies are required to capture the stakeholder’s perspectives which can highlight the challenges of implementing the recommendations of the current study. This stakeholder research should include parents, schoolteachers, school authorities and local administrators. A focus of the research should be on exploring parental influence on diet, family dietary traditions, and some solutions to the barriers highlighted in the present study. Additionally, intervention studies are needed to test the effectiveness of parent-focused education programs on reducing unhealthy food consumption in this cultural context. Furthermore, longitudinal studies could track how these barriers evolve with age.

## 5 Conclusion and recommendations

School lunch culture and practices in early childhood are not isolated entities but are constantly influenced by the various systems around them. The immediate environment, including family, school, peers, and community, significantly influences children’s food preferences, awareness of healthy eating, and ultimately their school lunch habits thereby shaping their daily experiences with food. Understanding these interconnected systems is crucial for comprehending individual food habits and creating supportive environments for healthy nutritional habits among schoolchildren in AD. The findings of the present study revealed that schools in AD have adopted policies and interventions targeting healthier school lunch with parent involvement and a supportive eating environment in schools. This has provided a foundation for developing effective, evidence-based policies that can impact the health and academic success of school children in AD as well as the UAE as a whole. To build on this, the significant influence of home environments should be recognized. Interventions should be developed to enhance nutritional literacy among parents, housemaids, and nannies encouraging healthier school lunch practices. Additionally, it is crucial to design culturally sensitive interventions that cater for the diverse children population in AD. This should ensure that school lunch menus are inclusive and appealing to all schoolchildren. Furthermore, creating a supportive school environment that allows sufficient time for eating school lunch is vital, as social interactions during meals can impact healthy food consumption.

## Data Availability

The raw data supporting the conclusions of this article will be made available by the authors, without undue reservation.
